# Hemichorea in 15-year-old patient with poorly controlled type 1 diabetes mellitus

**DOI:** 10.1186/1687-9856-2013-S1-P17

**Published:** 2013-10-03

**Authors:** Yong Hyuk Kim, Ho-Seong Kim

**Affiliations:** 1 Department of Pediatrics, College of Medicine, Yonsei University, Seoul, Korea

## Introduction

Hemichorea, spontaneous unilateral involuntary movements and contralateral neuroimaging abnormalities of the striatum may be the presenting feature of nonketotic hyperglycemia in older adults with type 2 diabetes, but cases in children with type 1 diabetes are very rare.

## Case

A 15-year-old woman with a 6-year history of type 1 DM developed righthemichorea. She presented continuous involuntary choreic movements of both herright arm and leg. The movements were nonsuppressible and ceased only during sleep.With the exception of this movement disorder, other neurological examination was unremarkable. On funduscopic examination, nonproliperative diabetic retinopathy was detected. The following laboratory findings were notable: fasting blood glucose 162 mg/dL, serum osmolarity 305 mOsm/L, and HbA1c 13.2%. Urinalysis was negative for glucose, ketones, and protein. There were no signs of diabetic ketoacidosis, hypoglycemia, hyperosmolar hyperglycemic coma, or rheumatic fever.Cranial computed tomographic scan showed that hyperattenuation of the left basal ganglia. T1-weighted magnetic resonance image demonstrated that hyperintensity of the left striatum.The hemichorea was slowly controlled with small oral doses of haloperidol (1.5 mg/d) and intensive blood glucose control.

## Conclusion

We report the case of a 15-year-old poorly controlled diabetic adolescent girl who developed acute hemichorea of the right arm and leg in whom T1-weighted magnetic resonance imaging of the brain revealed hyperintense signal in left basal ganglia.

